# Achieved negative differential resistance behavior of Si/B-substituted into a C_6_ chain sandwiched between capped carbon nanotube junctions†

**DOI:** 10.1039/d1ra08810f

**Published:** 2022-01-11

**Authors:** Najmeh Janatipour, Zabiollah Mahdavifar, Siamak Noorizadeh, Georg Schreckenbach

**Affiliations:** Department of Chemistry, Faculty of Science, Shahid Chamran University of Ahvaz Ahvaz Iran z_mahdavifar@scu.ac.ir +98-611-3331042; Department of Chemistry, University of Manitoba Winnipeg Manitoba R3T 2N2 Canada

## Abstract

Electronic transport properties of a pristine C_6_ chain and Si/B-substituted into the C_6_ chain sandwiched between two (5, 5) capped carbon nanotube electrodes were investigated through first-principles calculations based on non-equilibrium Green's functions (NEGF) conjugated with density functional theory (DFT). Si and B substitutions will affect the *I*–*V* curve of a pristine C_6_ chain. In the *I*–*V* characteristics, multi negative differential resistance (NDR) with large peak to valley ratio (PVR) and rectifying actions were observed. The NDR behavior originates from the joining and moving of conduction orbitals inside and outside of the bias window at a certain bias voltage. Furthermore, the assessment of transmission coefficient and distribution of molecular orbitals reveals that the rectifying performance is the result of the asymmetric distribution of the frontier molecular orbitals in the central region and their coupling with the electrodes. Multi NDR behavior of B substitution under very low bias voltage is a unique property of our proposed devices. Moreover, the CNT|C–(B–C)_2_–C|CNT molecular device shows a high PVR up to 31.8, which demonstrates that the proposed devices can be useful for molecular switching in nanoelectronic devices.

## Abstract 2

Multi negative differential resistance (NDR) with large peak to valley ratio (PVR) and rectifying actions were observed for a CNT|C–(B–C)_2_–C|CNT molecular device.

## Graphical abstract

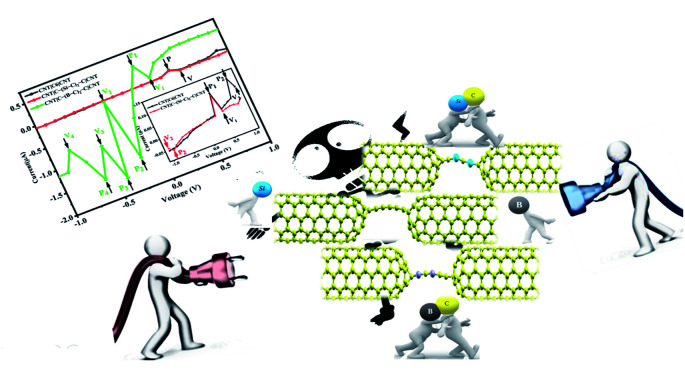

## 1. Introduction

The main idea of molecular electronics is to replace the traditional semiconductor-based components with single molecules to make nanoelectronic devices that are much smaller than those that existed in the past. In fact, the electronic transport properties of nanostructures have attracted much attention to the design of electronic devices at the nanoscale.^1–6^ Among carbon-based nanomaterials, low dimensional structures, specifically carbon nanotubes (CNTs) and quasi one dimensional (1D) atomic carbon chains, which were successfully carved out from graphene sheets using a high-energy electron beam,^7,8^ exhibit extraordinary electronic properties and also have played a prominent role in nanoelectronics.^8–10^ Carbon based nanoelectronic devices have interesting properties such as field-effect characteristics,^11–13^ switching behavior,^14,15^ rectification,^16,17^ and negative differential resistance (NDR).^8,9,18,19^ NDR behavior, which was first discovered in a tunnel diode by Leo Esaki,^20^ is described as increasing the applied bias voltage when the current decreases in the certain bias region. The NDR phenomenon is of great importance in the field of electronic technology, like electronic logic, amplification, and switching and memory circuits, due to low power dissipation, fast-speed operation, and high frequency characteristic in the negative differential resistors. NDR behavior in various systems including conjugate molecules, organic semiconductors and conductive polymers have been observed.^21,22^

The electronic properties of atomic carbon nanowires sandwiched between metal and carbon based electrodes were predicted theoretically.^8,9,14,18^ Khoo *et al.*^18^ reported the even–odd behavior and negative differential resistance (NDR) characteristic of linear atomic chains with 3, 4, 5, and 6 carbon atoms connected between two (5,5) capped CNT electrodes. The NDR effect was detected for both even and odd numbers of carbon atoms in the chains, although chains with an even number of carbon atoms carry current orders of magnitude smaller than that in the chains with an odd number of carbon atoms. Furthermore, Liew *et al.*^9^ investigated the electronic transport properties of different lengths of carbon atomic chains covalently connecting to a (5, 5) capped CNT and (3, 0) graphene nanoribbon as left and right electrodes, respectively. They realized that the rectifying performance was dependent on asymmetric distortion and movement of resonance while it is reduced when the chain states approach to the Fermi level. Moreover, Li *et al.*^23^ explored the electronic transport properties of linear atomic carbon chains, boron nitride chains and their heterojunctions connected between graphene electrodes. These systems exhibited even–odd behavior for longer lengths and current rectifying effect for even-numbered configurations except for the pristine carbon chains. Also, they found that the electronic properties of carbon and boron nitride chain heterojunctions can be modulated when the position and the number of boron nitride (BN) or carbon atoms changed. Furthermore, the current and the rectification ratio are enhanced for structures including carbon atoms in their junctions.

Three different hybridizations of carbon chains (sp, sp^2^, and sp^3^), can be included as important components of molecular devices due to the exceptional physical and chemical properties of atomic carbon chains. Cumulene with double bonds throughout the chain (=C=C=C=C=C=) and polyyne containing alternating single and triple bonds (–C≡C–C≡C–C≡) are considered as linear atomic carbon chains. Moreover, because of the small energetic stability difference between cumulene and polyyne, they could coexist under proper experimental conditions.^24^ It should be pointed out that the existence of sp chains at ambient conditions is still debated and challenged until today,^25^ however, synthesis of sp chains in its pure form has been carried out by cluster beam deposition.^26,27^ The experimental fabrication of free-standing linear carbon chains carved out from a graphene sheet has been achieved by high-energy electron beam.^7,28,29^ In addition to carbon chains, SiC chains^30^ possess unique physical and chemical characteristics in comparison with their bulk counterpart owing to quantum-size effects. 1D SiC nanowires have been experimentally synthesized,^31–33^ although it still remains a challenge to fabricate the ultimate one-atom thinness linear chains of SiC. It should be mentions that when contact is made with two metal electrodes, they can offer external strain to stabilize the chains. Furthermore, the possible existence of SiC chains has been predicted theoretically.^34^ The electron transport properties of atomic SiC chains,^35–37^ BN chains^23,38^ and also B or N substitution in (5,5) capped CNT electrodes^10^ have been investigated theoretically. Cheng *et al.* investigated the impact of (SiC)_*n*_ (*n* = 1–4) chain length for chains directly connected to Au electrodes.^35^ They found that the conductance of the SiC chain is highly sensitive to the local atomic arrangement, and also the length of the SiC chain is an important factor that affects their transport properties. Moreover, Mu *et al.* explored the electronic transport properties of two SiC chains sandwiched in parallel between two Au electrodes.^36^ Their results demonstrate that changing the separation between the two chains in a certain range can produce remarkable differences in transport properties. On the other hand, the electron transport properties of a single linear (SiC)_3_ chain inserted inside the hollow core of a (8,0) SiC nanotube connecting between two Au electrodes were investigated by Mu *et al*.^37^ They showed that the encapsulation can evidently modify the electronic structure and transport properties of the isolated (8,0) SiCNT and the SiC chain.

In this study, we have investigated the electron transport properties of molecular devices constructed from different atomic carbon chains consisting of six carbon atoms (C_6_), Si-substitution into the carbon chain C–(Si–C)_2_–C, and B-substitution into the carbon chain C–(B–C)_2_–C connected between two (5,5) capped carbon nanotube electrodes. It should be pointed out that a 1D boron carbide (BC) nanowire has been synthesized experimentally and several methods are available to produce different morphological structures of BC chains.^39–41^ Our proposed molecular devices display NDR behavior with large peak-to-valley ratio and obvious rectifying performance. The NDR behavior originates from the conduction orbitals being joined and shifted inside or out of the bias window at the certain bias. Furthermore, the rectifying performance of our proposed molecular devices is derived from the asymmetry distribution of the frontier molecular orbitals (FMO) plus their coupling to the CNT electrodes which could be proved through the assessment of transmission coefficient and distribution of molecular orbitals (MO). Our proposed devices show a high peak to valley ratio (PVR) up to 31.8 for the B dopant. These results demonstrate that our proposed devices are useful for molecular switching, diodes, logic, multipliers, high-frequency oscillators and other practical applications.

## 2. Models and methods

First-principles calculations based on density functional theory (DFT) conjugated with non-equilibrium Green's functions (NEGF)^42^ implemented in the TranSIESTA^42,43^ package were performed to evaluate the electronic structures and electronic transport properties of CNT|C_6_|CNT, CNT|C–(Si–C)_2_–C|CNT and CNT|C–(B–C)_2_–C|CNT molecular devices. In the theoretical approach presented in this research work, the exact expression for the current within the NEGF framework and employing a strong-coupling cluster approximation to evaluate the required Green's functions (GFs) was used. Hence, all interactions of the nanoscale device be it of electron–electron or electron–phonon nature have been exactly taken into account.^44–46^ The carbon chain was modeled by the H_C_ = H_e_ + H_p_ + H_ep_ Hamiltonian. The first term, H_e_, qualifies the motion of the electrons, the second term H_p_ is the vibrational part, and the last term of the carbon chain Hamiltonian incorporates the Su–Schrieffer Heeger interaction between the electrons and the dynamic phonons.^47^ Due to the Hamiltonian of the molecule, the effect of molecular vibration is included in the Hamiltonian. The Troullier–Martins norm-conserving pseudopotentials^48^ were used to describe the core electrons. The PBE parameterization of the generalized gradient approximation (GGA) was used to describe the exchange-correlation functional. Additionally, the double-*ζ*-plus polarization (DZP) basis set has been used to describe the crystal orbitals. An energy cutoff of 150 Ry was chosen as a required energy for the grid integration in the real space. The *k*-point sampling 1 × 1 × 4 was used in the *x*, *y*, *z* directions, respectively (see Fig. S1†) and a 1 × 1 × 100 *k*-point mesh was used for the self-energy calculations of the electrodes. The energy convergence was set to 10^−5^ eV and the atomic positions were fully relaxed until the atomic forces were smaller than 0.02 eV Å^−1^. The electronic temperature is 300 K in the Fermi function. The device current (*I*) through the central scattering region can be calculated by the Landauer–Büttiker formula^49^ at finite bias as follows:1
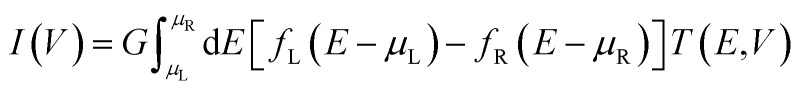
where *G* = 2*e*/*h* is the quantum unit of conductance, and *e* and *h* are electron charge and Planck's constant, respectively. The Fermi function of the left (right) electrode is *f*_L(R)_ = [1 + *e*^(*E*−*μ*_L(R)_)/*k*_B_*T*^]^−1^ with *μ*_L(R)_ being the electrochemical potential of the left (right) electrode. *T*(*E*, *V*) is the transmission probability of electrons with energy *E* for bias voltage *V*. The transmission coefficient of the device can be determined as:

2 *T*(*E*, *V*) = Tr[*Γ*_L_(*E*, *V*)*G*^R^(*E*, *V*)*Γ*_R_(*E*, *V*)*G*^A^(*E*, *V*)]

where *G*^R^ and *G*^A^ are the retarded and advanced Green's functions, and *Γ*_L/R_ are the broadening functions which are the imaginary parts of the left and right electrodes, respectively.

## 3. Results and discussion

**Fig. 1:**
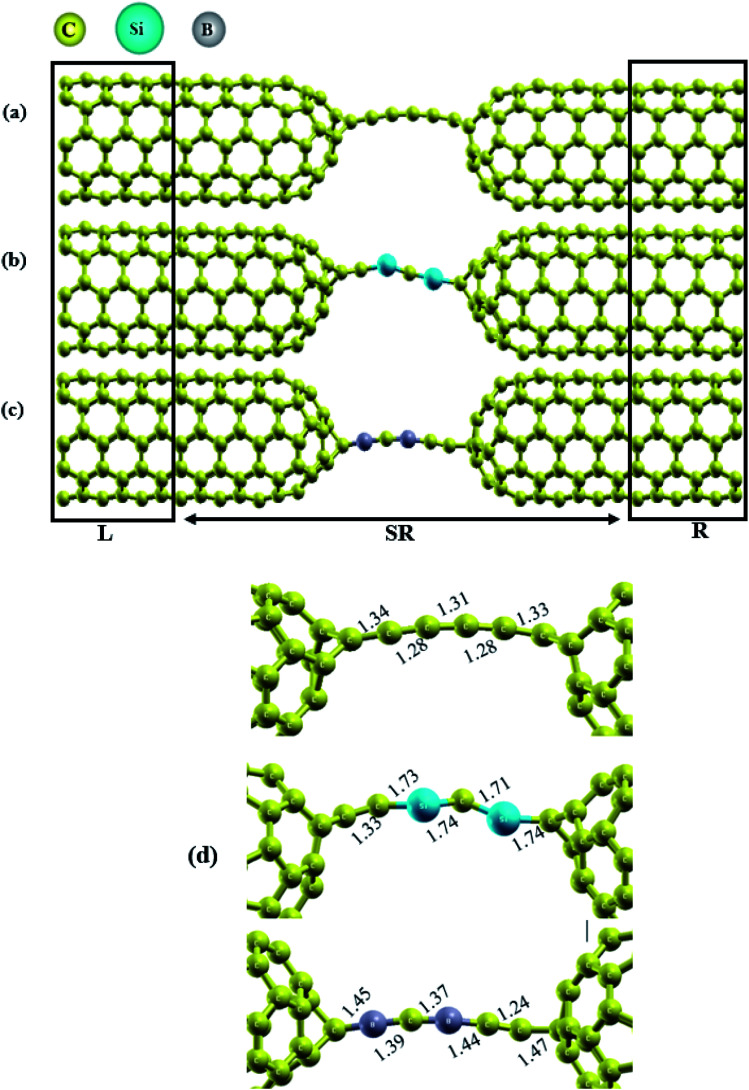
Final relaxed geometries of (a) C_6_, (b) C–(Si–C)_2_–C and (c) C–(B–C)_2_–C junctions with CNTs electrodes. The number of atoms in each chain is 6. The labels L, SR and R denote the left electrode, the scattering region, and the right electrode, respectively. In (d), the C–C, Si–C and B–C bond lengths (Å) in each chain are represented.

Herein, we investigated the behavior of Si/B-substituted into a C_6_ chain sandwiched between capped carbon nanotubes. The final relaxed geometries of the molecular transport devices are represented in Fig. 1. As seen from this figure, the CNT|C_6_|CNT, CNT|C–(Si–C)_2_–C|CNT and CNT|C–(B–C)_2_–C|CNT molecular devices can be divided into three regions: left electrode, central scattering region and right electrode. For simplicity, we named the devices based on their carbon chains hereafter, *i.e.*, as C_6_, Si–C, and B–C junctions. As shown in Fig. S1,† the initial distance between the electrodes for pristine, B- and Si-substituted chains is about 7.0, 7.2 and 8.1 Å, respectively, which is in good accordance with the pervious theoretical studies.^15,18^ It should be noted that after geometry relaxation, these distances were changed (in the range of 6.5–8.4 Å). As shown in Fig. 1, the molecular device was divide into three regions: (1) the central region, (2) the left, and (3) the right metallic leads. The lengths of the central region for the devices containing C_6_, Si–C and B–C chains are 26.8, 27.9 and 27.6 Å, respectively, which indicates that the distances between the two electrodes are nearly the same. Furthermore, the polarization of the bonds can exist in the junction when it is doped at other sites or in terminal-substituted carbon chains. Hence, to reduce this effect we consider the doping effects of Si and B atoms onto the carbon chain. We are assuming that one carbon atom each at the two ends of the caps is covalently bridged in the left and right caped CNTs just with carbon atoms of chain which means carbon atoms serving as anchor atoms. These constructed models are represented in Fig. S1.† It should be noted that the initial angle between the electrodes and chains is fixed at 180°. Then we consider the different substitutions as displayed in Fig. S11(b and c):† two Si/B atoms were substituted for two carbon atoms at the middle of the carbon chain, so that, after these substitutions, the carbon atoms at both ends of the chain were covalently bonded to the electrodes with an angle of 180° (see Fig. S1†).

The scattering region for each device consists of the C_6_, C–(Si–C)_2_–C or C–(B–C)_2_–C junction (6 atoms) and the (5,5) capped CNTs which are derived from half of a C_60_ molecule within five nanotube lead layers on either side of the junction (comprising 160 carbon atoms) to consider the molecule-electrode coupling. The remaining left and right electrodes are composed of six (5, 5) CNTs layers (60 carbon atoms each). The total number of atoms are 280 + 6 in each device, where the number 6 refers to the atoms in the junction proper. We notice that our CNT|C_6_|CNT model has 60 carbon atoms more than the model of Khoo *et al.* for the C_6_ chain.^18^ As the electronic structure of the electrodes is of great importance for the interoperation of the device transmission spectra, the electronic band structure of the (5, 5) CNTs electrodes has also been investigated. The CNTs have shown excellent electronic properties which is useful in nanoelectronic devices applications. On the other hand, carbon nanotubes have high electrical conductivity which is comparable to copper. Especially notable is the fact that nanotubes can be metallic or semiconducting. Our calculations predict that the (5, 5) CNTs electrodes show metallic behavior with zero band gap. As can be seen in Fig. S2,† the valence band maximum (VBM) and the conduction band minimum (CBM) meet each other at the Fermi energy which means that the (5, 5) CNT is a metallic one. Therefore, due to the electrical properties, high symmetry, as well as the appropriate diameter size (*d* ≈ 7 Å), this nanotube has been selected as electrodes in this research work.

To investigate the effect of substitution atoms on the electronic transport properties, two carbon atoms in the C_6_ junction were replaced by B or Si atoms. Furthermore, the two ends of the carbon chain are covalently bonded to equivalent atoms on the tip pentagons in each capped CNT electrode. This is one of the advantages of using capped-CNTs as electrodes in devices. The two carbon atoms at the end of the chain junctions were fixed, and the other four carbon atoms of the chain were alternately replaced by B and Si atoms to form C–(Si–C)_2_–C and C–(B–C)_2_–C junctions, respectively. It is well known that the electronic transmission and transport features of molecular devices can be affected strongly by the electronic structure of the chains.^6,7,10,35,50,51^

The relaxed geometries of the carbon chains are shown in Fig. 1(d). The C–C bond lengths in the pristine carbon chain are alternating between 1.28 and 1.34 Å for single and triple bonds, respectively, demonstrating distinct polyyne character in the ground state. Also, these results are in good accordance with previous studies.^6,7,18,50^ In the final relaxed geometry of the molecular devices (Fig. 1), the carbon chain connected between two capped CNTs electrode acquires a slightly bent geometry.^9,17,18^ In the case of substitution chains, the Si–C bond lengths in the C–(Si–C)_2_–C chain are in the range of 1.71–1.74 Å which is significantly larger than the theoretical Si–C bond length of 1.65 Å for the independent chain,^52^ indicating that the distance between the C and Si atoms is affected by the surrounding materials. It should be noted that no symmetry was observed on either side of the C–(Si–C)_2_–C chain. For the C–(B–C)_2_–C chain, our calculations result in optimized B–C bond lengths of about 1.37–1.45 Å. Except for the right side of the CNT|C–(B–C)_2_–C|CNT device, the five-membered rings located at the end of the CNT cap in other devices were transformed into six-membered rings, induced by a broken bond in the cap and junctions (see Fig. 1(d)). These observations are also in accordance with previous studies. As a consequence, the carbon chain acquires a slightly bent conformation in the three different junctions.^9,18^ It is obvious from the relaxed geometries that the three junctions show asymmetric behavior due to the asymmetry in the molecular chains.

**Fig. 2:**
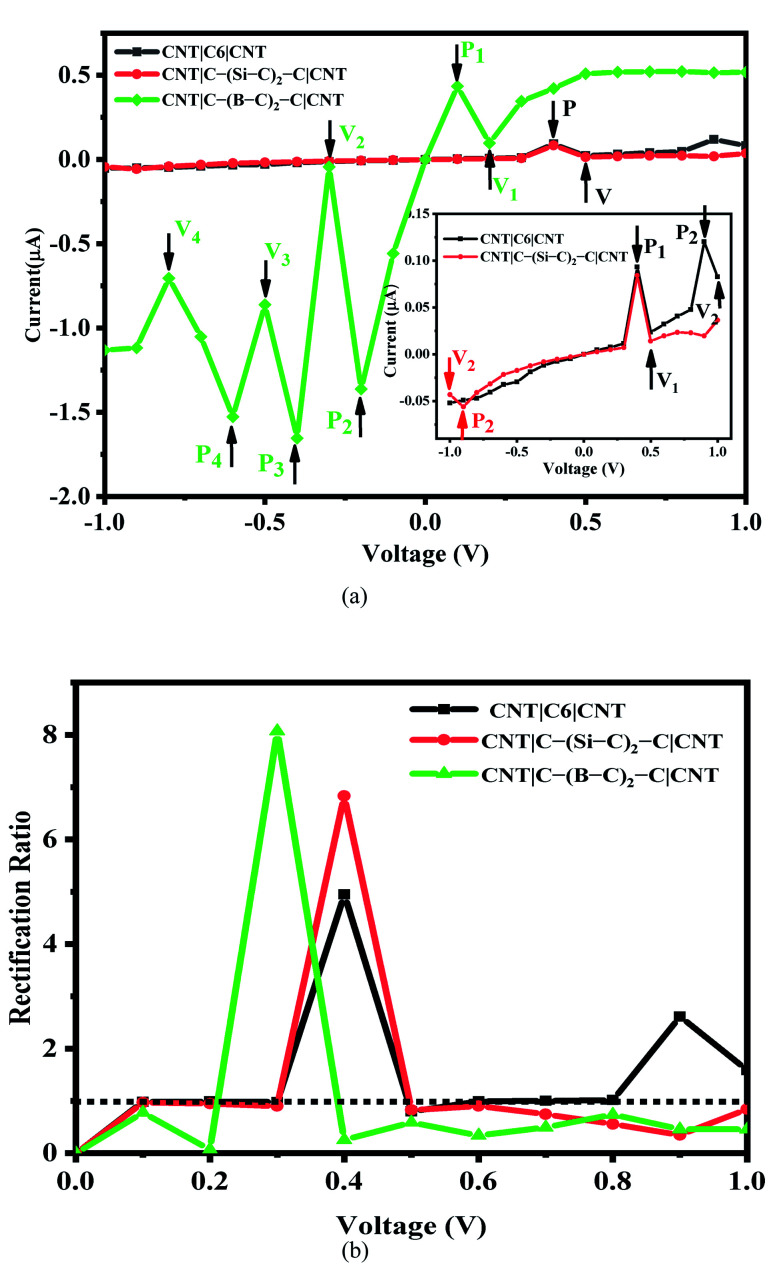
(a) The *I*–*V* curves and (b) rectification ratio change with various bias voltages of C_6_, C–(Si–C)_2_–C and C–(B–C)_2_–C junctions. P_*i*_ and V_*i*_ (*i*=1, 2, 3, 4) indicate the peak and valley positions for each junction. The dotted line indicates *R*(*V*) = 1.

The current–voltage (*I*–*V*) characteristics and rectification ratio of the C_6_, Si–C and B–C junctions under a bias voltage range from −1 to 1 V has been investigated. The results are shown in Fig. 2(a). As seen from this figure, the *I*–*V* curves of the pristine C_6_ and Si–C chains are exhibiting double negative differential resistance (NDR) characteristics. The first NDR appears when the bias voltage (*V*_b_) is increased from 0.4 to 0.5 V for both the C_6_ and Si–C junctions. These results are in good accordance with other systems^17,53^ such as carbon chain with a phenyl ring,^14,54^ Na atomic chain,^55^ and the Si monoatomic chain.^56^ In comparison, for the Au|SiC|Au device,^35^ no NDR effect was observed due to top–top geometry contact,^57^ while the NDR effect is common in CNT-based junctions.^9,17,18^ This result shows the effect of the electrode in the molecular devices. The second NDR of the C_6_ junction occurs at higher positive voltage from 0.9 to 1 V and it is slightly sharper than the first NDR. Compared with the C_6_ junction, the *I*–*V* cure of the Si–C junction shows a smaller second NDR effect at high negative voltage from −0.9 to −1 V. It is worth mentioning that the current is increasing at the bias of 0.9 V which may be the start of a new NDR effect for the Si–C junction (see Fig. S3b†). Furthermore, the currents of the pristine C_6_ junction are slightly greater than those of the Si–C junction in the positive bias voltage range and for several negative bias voltages. In the case of the B–C junction, the calculated currents are much stronger than those of the C_6_ and Si–C junctions over the entire bias voltage range. This may be related to the more extended π electron network of the B–C junction leading to a higher current ratio. These results are plotted in Fig. 2.

The maximum value of current for the first (second) NDR effect is ∼0.093 (0.120) μA, which is observed at 0.4 (0.9) V for the pristine C_6_ junction. On the other hand, the current reaches to a minimum value of about 0.024 (0.083) μA, at the valley site with *V*_b_ = 0.5 (1.0) V. For the Si–C junction, the first (second) peak current is 0.084 (0.056) μA and the valley current reaches to 0.014 (0.043) μA at 0.5 (−1.0) V. As can be seen from Fig. 2(a), the position of the peaks and valleys in each *I*–*V* curve are determined by P_*i*_ and V_*i*_ (*i* = 1, 2, 3, 4). The *I*–*V* characteristic of the B–C junction exhibits obvious multi-NDR behavior at negative and low positive bias voltage. Overall, for the B–C junction, three regimes with clear NDR effects exist between −0.2 to −0.8 V, which are represented by P_2_, P_3_, P_4_ and V_2,_ V_3,_ V_4_, plus a NDR regime between 0.1 to 0.2 V (P_1_ and V_1_), as shown in Fig. 2(a). The first current peak (*I*_peak1_) for positive bias voltage is observed at 0.1 V and the first valley current (*I*_valley1_) appears at 0.2 V. For the negative bias voltage, the *I*–*V* curve characteristic of the B–C junction shows triple NDR effects, Fig. 2(a). The current increases at −0.2 V to the second current peak (*I*_peak2_) and the second current valley (*I*_valley2_) appeared by decreasing the current at voltage −0.3 V. Also, at −0.4 V, the current increases again to the third current peak (*I*_peak3_) and then at −0.5 V the current decreases to the third current valley (*I*_valley3_). Finally, the current increases to the fourth current peak (*I*_peak4_) at −0.6 V and then decreases to the forth current valley (*I*_valley4_) at −0.8 V. The values of current at the peaks are 0.434, 1.362, 1.652 and 1.526 μA, corresponding to P_1_, P_2_, P_3_ and P_4_, respectively, whereas the magnitudes of the valley currents are obtained as 0.098, 0.043, 0.861 and 0.704 μA for V_1_, V_2_, V_3_ and V_4_, respectively. As a result, the B–C junction in comparison to the Si–C junction has a dramatically increased current. The B–C junction has a maximum *I*_peak3_ value of 1.652 μA, which is considerably larger than that of the other junctions. The inequivalent and variation coupling of C_6_, Si–C, and B–C molecular orbitals with incident states from contacts and CNT electrodes is a possible mechanism to explain the NDR behavior which results in different charge effects and also different currents.

To describe the NDR effects, the current peak to valley ratio (PVR = *I*_peak_/*I*_valley_) is defined. The PVR1 and PVR2 for the pristine C_6_ (Si–C) junction are calculated to be 3.95 (5.90) and 1.45 (1.29), respectively. In the case of the B–C junction, there are four different PVR that can be calculated. The PVR2 value of the B–C junction is the largest on-off peak-to-valley ratio with a value in excess of 31.82, which is larger than the PVR value of the prototypical solid-state GaAs/AlGaAs Esaki-diode.^20^ Other PVR values for the B–C junction are 4.43 (PVR1), 1.92 (PVR3), and 2.17 (PVR4), respectively. The higher PVR value and multi-NDR effects of the B–C junction in comparison with the C_6_ and Si–C junctions enhance the potential for applications of utilizing NDR for electronic devices.

As seen from Fig. 2(a), the *I*–*V* curves of the investigated molecular devices show an asymmetric behavior which is related to the asymmetry in the C_6_, Si–C, and B–C chains as well as the asymmetric coupling of the chains to the CNTs electrodes. The asymmetric behavior of the *I*–*V* curves can be described by the rectification ratio which is the ratio of currents under negative and positive bias voltage for the same voltage magnitude, defined by the following expression:

3 *R*(*V*) = *I*(+*V*)/|*I*(−*V*)|

*R*(*V*) > 1 indicates forward rectifying behavior, *R*(*V*) = 1 represents no rectification, and *R*(*V*) < 1 corresponds to reverse rectifying behavior. Based on our calculations (see Fig. 2(b)), the B–C junction has the strongest rectification ratio, reaching to 8.1 at 0.3 V, which is forward rectifying behavior. In the case of Si–C and C_6_ junctions the rectification values obtained are about 6.8 and 4.9 at 0.4 V, respectively. Furthermore, the C_6_ junction shows a rectification behavior (2.61) at 0.9 V. It should be noted that the B–C junction also shows a reverse rectifying behavior at *V*_b_ < 0.2 V and *V*_b_ > 0.4 V.

**Fig. 3:**
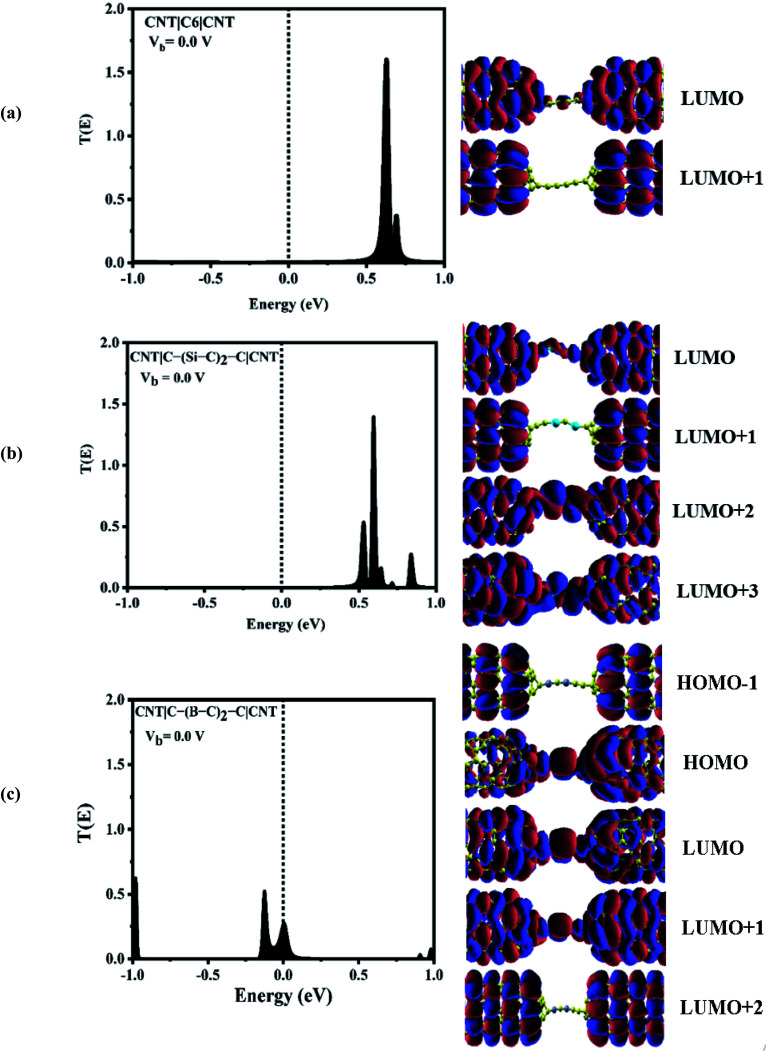
Left: transmission spectra and spatial distribution of orbitals for (a) CNT|C_6_|CNT, (b) CNT|C–(Si–C)_2_–C|CNT, and (c) CNT|C–(B–C)_2_–C|CNT molecular devices at zero bias. The dashed lines represent the Fermi level. Right: frontier molecular orbitals.

To explore the physical basis of the NDR effects and rectifying performance, the energy-dependent transmission spectra (T(E)) of the three molecular junctions were also investigated. The transmission value is defined as the degree of delocalization of the molecular orbitals and hybridization of wave functions and the probability of transmitting an electron from the first electrode to the second electrode in the scattering region. According to the Landauer–Büttiker formula,^49^ the current is obtained by the integration of the transmission coefficients in the bias windows (−V/2 to V/2). It depends on the magnitude of the bias window and the transport coefficient. To better understand the electron transport behavior of the molecular devices, the transmission spectra of the C_6_, Si–C and B–C molecular junctions at zero bias have been calculated, Fig. 3. In order to assign a large number of electronic resonances, the energy range of −1 eV to +1 eV with respect to the Fermi energy of the junctions was considered. The main important features of the transmission spectra at zero bias are resonance peaks at around the Fermi energy in electron transport systems. The prime transport channels for the C_6_, Si–C and B–C molecular junctions are the lowest unoccupied molecular orbital (LUMOs) as shown in Fig. 3 which shows both the transmission spectra as well as the corresponding spatial distributions of the LUMO and LUMO+1 peaks for the C_6_ junction, LUMO, LUMO+1, LUMO+2, and LUMO+3, resonance peaks for the Si–C junction and finally HOMO−1, HOMO, LUMO, and LUMO+1 peaks for the B–C molecular junction at zero bias. The conduction of electrons through the molecular chain attached to the contacts can be explained *via* the shape of the FMOs. When these FMOs are fully delocalized over the whole devices, they possess a greater transmission probability.

Fig. 3 (a) shows that there are sharp LUMO and weaker LUMO+1 peaks for the C_6_ molecular junction at energies of 0.630 and 0.690 eV, respectively. On the other hand, for the Si–C device, the LUMO+1 peak is heightened at an energy of 0.595 eV while the peaks of LUMO, LUMO+2 and LUMO+3 appear at energies of 0.535, 0.64, and 0.71 eV, respectively (see Fig. 3(b)). These MOs cannot be excited to transport electrons under very low positive bias owing to the fact that they are far away from the Fermi level. Therefore, there is a very small current under the negative and high positive bias voltage regime for the C_6_ and Si–C junctions. The LUMOs, except for the LUMO+1, are spread over the whole scattering region which including the chain and the central part of the CNTs for the C_6_ and Si–C junctions. Effective coupling of the FMOs of chain and electrodes determines the intensities of the resonance peaks. In comparison, the LUMO (LUMO+1) peak of the C_6_ (Si–C) junction has the highest transmission coefficient because it is delocalized over the whole scattering region (distributed over the whole CNT electrodes). The LUMO+3 is more delocalized on the Si–C chain and the left CNT lead which is indicative of the electronic coupling strength of the left CNT's lead with the molecular states of the Si–C chain. Furthermore, the delocalization of the LUMO+3 is decreased by moving to the right lead (see Fig. 3). On the other hand, in the case of the B–C molecular junction, two broad resonance peaks at the Fermi energy appear at energies of −0.126 and 0.006 eV indicating the metallic nature of the CNT|C–(B–C)_2_–C|CNT device. As seen from Fig. 3(c), the LUMO peak shifts down further and passes the Fermi energy to become the HOMO; hence HOMO and LUMO are the dominant transport resonances. Also, HOMO−1 with a high transmission coefficient appears at lower energies while LUMO+1 and LUMO+2 with small transmission coefficients are located at higher energies. The HOMO−1 is localized over the whole CNT electrodes and the delocalization of HOMO increases by moving to the right CNT electrode which is related to much stronger coupling of the B–C molecular states with the right CNT's lead. In addition, the LUMO and LUMO+1 distribute over the whole molecular scattering region leading to the appearance of resonance peaks at the Fermi level.

**Fig. 4:**
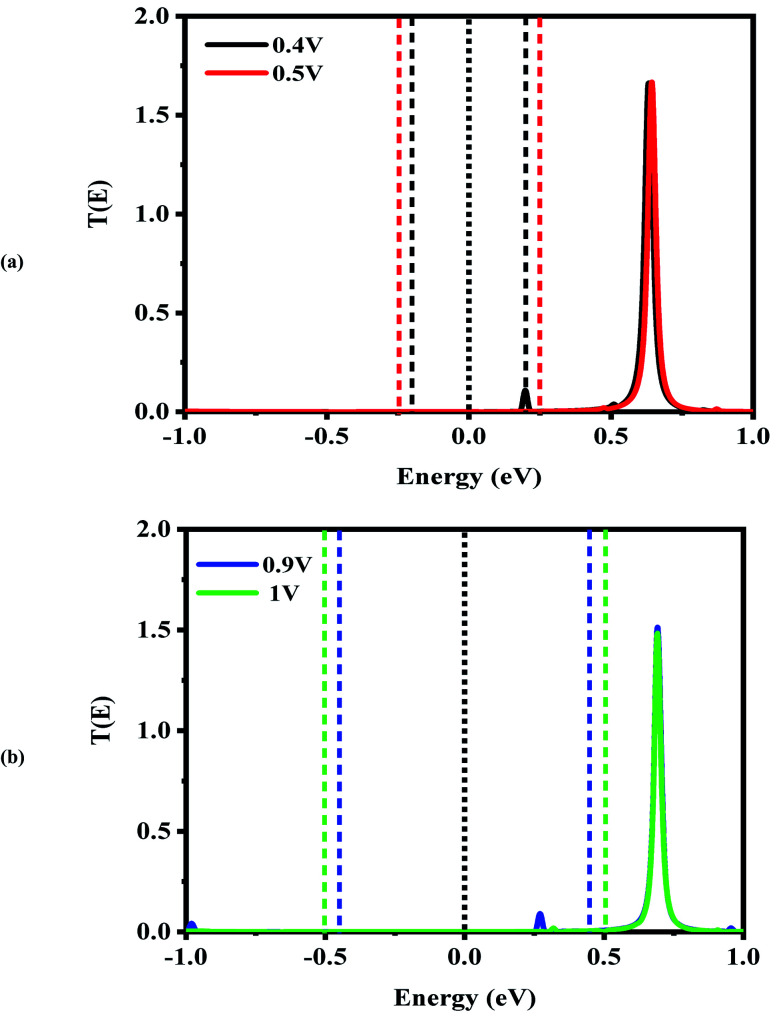
Bias-dependent transmission spectra of the CNT|C_6_|CNT molecular device at different voltages, (a) 0.4 and 0.5 V and (b) 0.9 and 1.0 V. The Fermi level (black dotted line) is set to zero and the energy region between the two dashed lines shows the bias window.

**Fig. 5:**
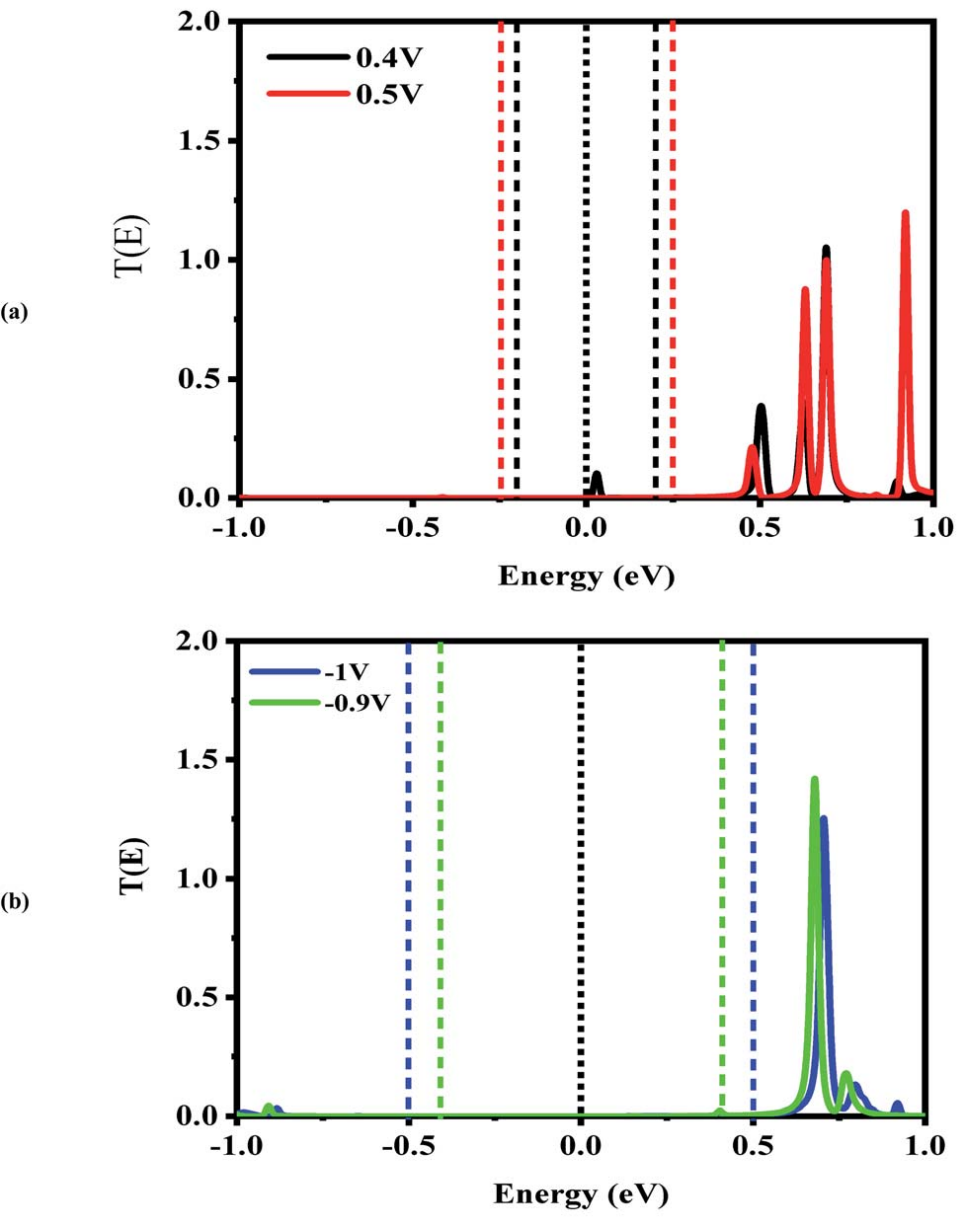
Bias-dependent transmission spectra of the CNT|C–(Si–C)_2_–C|CNT molecular device at different voltages, (a) 0.4 and 0.5 V and (b) −0.9 and −1.0 V. The Fermi level (black dotted line) is set to zero and the energy region between the two dashed lines shows the bias window.

**Fig. 6:**
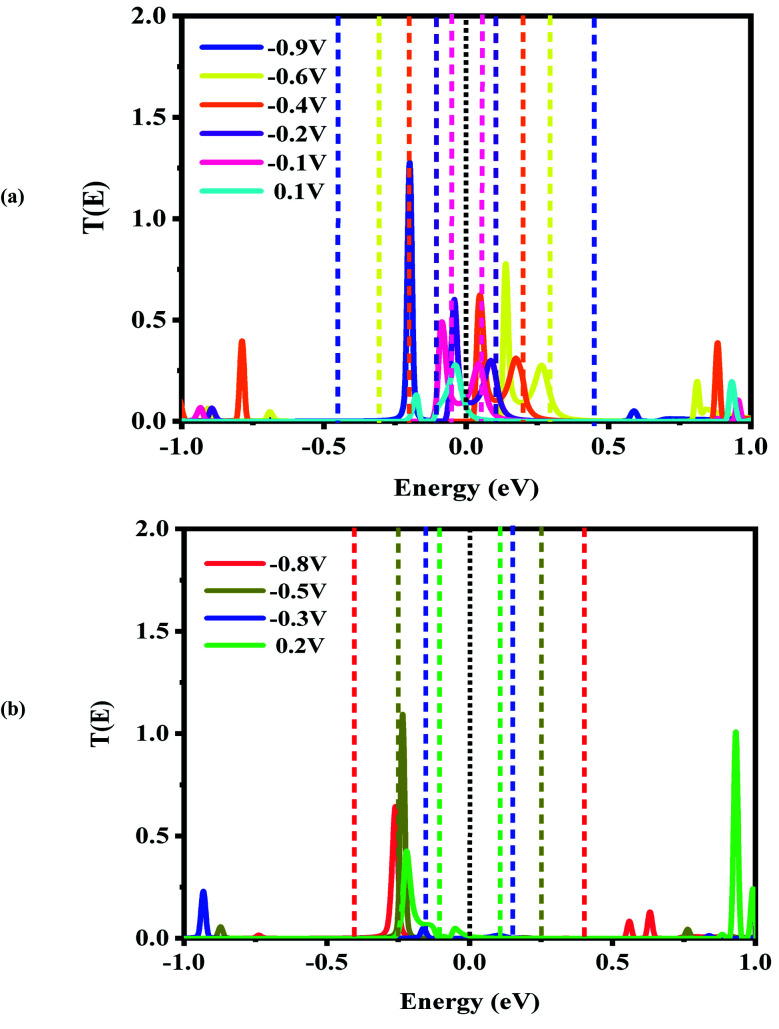
Bias-dependent transmission spectra of the CNT|C–(B–C)_2_–C|CNT molecular device at different voltages, (a) −0.9, −0.6, −0.4, −0.2, −0.1 and 0.1 V and (b) −0.8, −0.5, −0.3 and 0.2 V. The Fermi level (black dotted line) is set to zero and the energy region between the two dashed lines shows the bias window.

Next, the nonequilibrium transmission spectra of the molecular devices were investigated. They are presented in Fig. 4–6. As shown in Fig. 4, for the CNT|C_6_|CNT system, with increasing the positive bias, although the bias window constantly expands, the current increases. As a result, the *I*_peak_ appears at a voltage of 0.4 V due to the LUMO peak located on the edge of the bias window. It should be noted that it does not enter the bias window while it shifts out of the bias window at −0.4 V which is represented in Fig. S3(a),† and also shifts to the higher energies with respect to the Fermi level. When the bias voltage goes to values higher than 0.4 V, the current decreases and the *I*_valley_ is appearing because the LUMO and LUMO+1 peaks merge into a sharp and high peak at the bias of 0.5 V which is located far away from the Fermi level. As seen in Fig. S3(a),† two separated resonance peaks are joined to each other with an increase in the bias voltage. They are again separated at a voltage of 0.6 V. The second NDR effect is detected by enlarging the bias window. Therefore, the LUMO peak enters the bias window again causing an increase in the current when the bias reaches to 0.9 V. For 1 V, the current drops due to the transmission value of the LUMO peak at the bias window. It is obvious that the transmission coefficient is very low and far away from the Fermi level at 0.4 V and 0.9 V which leads to small amounts of charge transport.

In comparison with the CNT|C_6_|CNT system, it is found from Fig. 5 that the LUMO peak of the CNT|C–(Si–C)_2_–C|CNT transport molecular system shifts to lower energies with increasing the bias voltage and finally enters into (out of) the bias window at 0.4 V (−0.4 V as shown in Fig. S3(b)).† More importantly, the LUMO peak sticks to the Fermi level, and hence the current is increased at 0.4 V, while it leaves the bias window and shifts to higher energies at 0.5 V. Therefore, the current will drop while the transmission coefficients of LUMO+1 and LUMO+2 are increased. Overall, Fig. S3(b)† indicates that the LUMO shifts to lower energies with increasing the bias voltage but it is located at the edge of the bias window and moves into the bias window at −0.8 V. Finally, the current reaches to larger (smaller) value at −0.9 V (−1.0 V) owing to the fact that the LUMO with low transmission coefficient is at the edge of (outside of) the bias window; hence, the second NDR can be observed. It can be expected that the current slightly increases at these voltages which is in accordance with Fig. 2(a). It is worth mentioning that the LUMO+1 and LUMO+2 peaks are joined to each other at higher positive voltage. The transmission peaks in the range of 0.7–1 eV appear to be due to the charge transients from LUMO+2, LUMO+3, and LUMO+4, respectively. The transmission calculations for the C_6_ junction indicate that LUMO+1 at a bias of 0.4 V and 0.9 V has a higher transmission coefficient than the LUMO peak which is responsible for the rectifying performance, and it has less contribution to transport. This is similar to the Si–C junction where the main contributing orbital at 0.4 V (−0.9 V) is the LUMO peak while the LUMO+2 (LUMO+1) has the highest transmission coefficient.

**Fig. 7:**
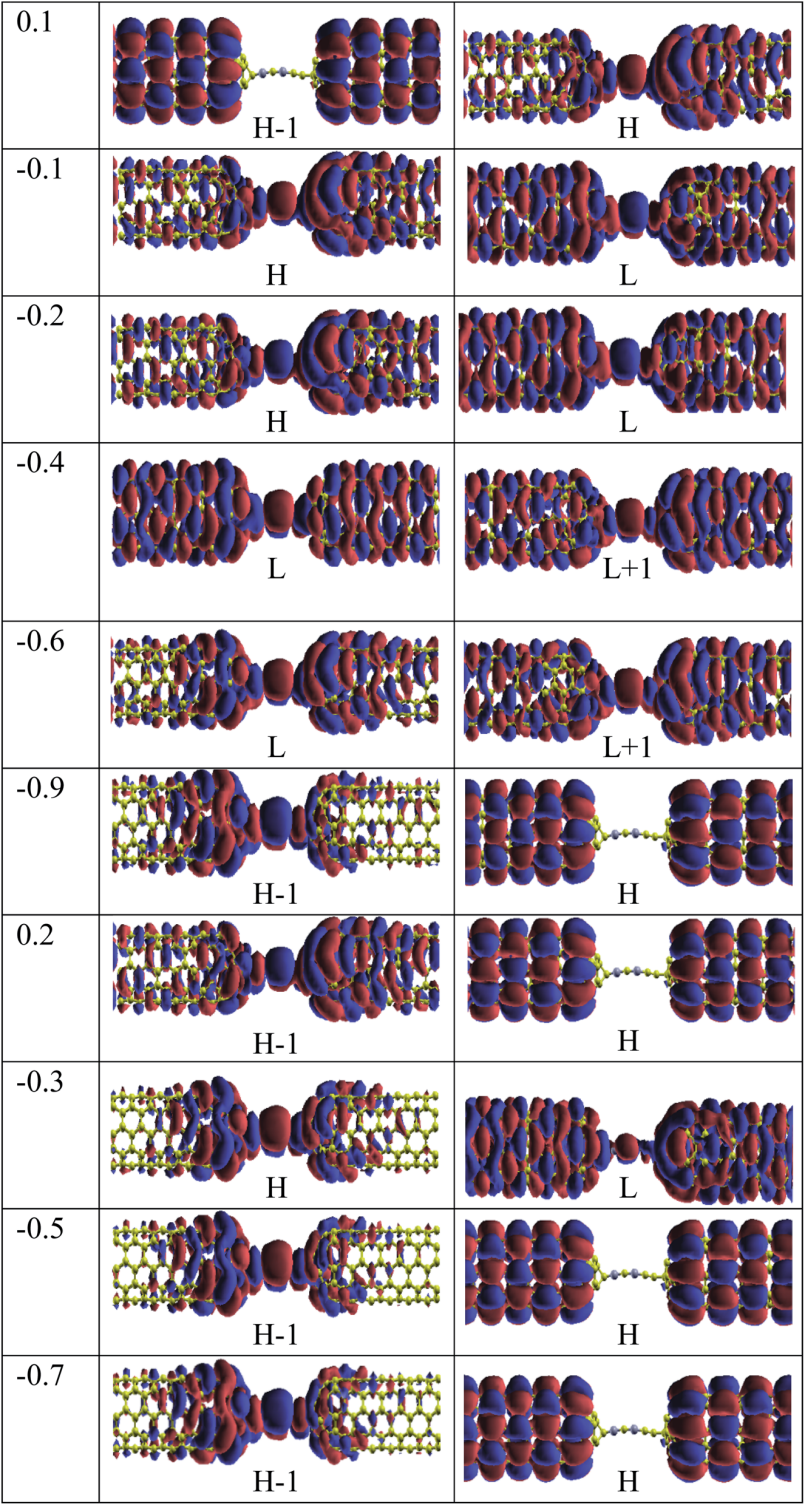
Frontier molecular distributions of CNT|C–(B–C)_2_–C|CNT device at various bias voltages.

For the CNT|C–(B–C)_2_–C|CNT transport system, the bias dependent transmission spectra are represented in Fig. 6, and the FMO distributions are summarized in Fig. 7. The resonance peaks (from the first *I*_peak1_ to the last *I*_peak4_) shift to energies below the Fermi level when the bias voltage is applied. This can be compared to the equilibrium resonance peaks which are located at the Fermi level (see Fig. 6 (a)). The resonance peaks pass the Fermi energy to become the lowest unoccupied molecular orbitals. By enlarging the bias windows, two transmission resonance peaks are fully located within the bias window leading to a significant increase in the current. Moreover, the transmission coefficients of the LUMO peaks will be increased with increasing bias voltage, and these peaks move to energies above the Fermi level. Based on our calculations, the maximum value of peak current belongs to resonance peaks at −0.4 V which is corresponding to the delocalization of LUMO and LUMO+1 (Fig. 7). Also, HOMO−1 and LUMO+2 at −0.4 V are located far away from the Fermi energy, with higher transmission coefficients than the other biases. At the bias −0.9 V, two resonance peaks are joined to each other and shift to lower energies. The current is increased when the HOMO peak is heightened at −0.9 V due to increased delocalization of the HOMO over the whole CNTs (see Fig. 7).

Based on Fig. 6(b), two resonance peaks move to energies below the Fermi level and merge into a sharp transmission peak at higher negative voltage. For 0.2 V, the first valley current appears when two resonance peaks pass the Fermi energy to become HOMO and HOMO−1 peaks but only the HOMO peak with low transmission coefficient is located in the bias window causing reduced current. As seen, the second valley current appears at −0.3 V. The HOMO peak becomes manifest at the edge of the bias window while the LUMO enters into the bias window. The transmission coefficients are reduced severely and the minimum valley current *I*_valley2_ is exhibited. The HOMO (LUMO) delocalization increases by moving to the left (right) electrode. These two resonance peaks are responsible for the rectifying performance although they have very low transmission values. Furthermore, one HOMO peak with higher (lower) transmission value is detected at the edge of the bias window (inside the bias window) for −0.5 V (−0.8 V) which results in the current *I*_valley3_ (*I*_valley4_). Only for 0.2 V, there are significant transmission coefficients of LUMO and LUMO+1, at 0.92 eV and 0.99 eV, respectively.

As is obvious from Fig. S3(c),† the overall shapes of the transmission resonance peaks are similar (for positive bias from 0.3 V to 0.8 V) to those at a bias of 0.3 V. These peaks move to slightly lower energies, while the HOMO stays inside the bias window due to its enlargement. This behavior results in approximately equivalent currents for biases of 0.4 V to 1 V. This results in the multi NDR effect for the CNT|C–(B–C)_2_–C|CNT transport system at negative bias voltage which is derived from the HOMO and LUMO levels. Comparing the three different systems, the HOMO and LUMO levels of the CNT|C–(B–C)_2_–C|CNT molecular device are located at the Fermi level while the LUMO levels for the CNT|C_6_|CNT and CNT|C–(Si–C)_2_–C|CNT transport system are farther from the Fermi level. Thus, the double NDR effect is to be expected.

## 4. Conclusion

The electronic transport properties of pristine, Si- and B-substituted into the carbon chains consisting of six atoms bound to (5,5) capped CNTs electrodes were investigated using non-equilibrium Green's functions (NGEF) in conjunction with density functional theory (DFT). The current is strongly affected by Si- and B- substitutions. The CNT|C–(B–C)_2_–C|CNT device has higher currents than the pristine CNT|C_6_|CNT and CNT|C–(Si–C)_2_–C|CNT transport systems. Also, the asymmetric contacts and geometries of the molecular junctions result in asymmetric *I*–*V* curves. Multi-NDR behavior at very low positive bias and along negative biases is observed for the B–C junction, while the C_6_ and Si–C junctions display double-NDR behavior. The NDR behavior arises from joining and moving conduction orbitals inside and outside of the bias window. The asymmetry distribution of the FMOs in the central region and their coupling to the electrodes result in largest peak-to-valley and rectification ratios upon B substituted into the C_6_ chain. These results demonstrate that our proposed devices are useful for molecular switching, diodes, logic, multipliers, high-frequency oscillators, and other practical applications.

## Conflicts of interest

There are no conflicts to declare.

## Supplementary Material

RA-012-D1RA08810F-s001
