# Supplementary material for: Achieved negative differential resistance behavior of Si/B-substituted into a C6 chain sandwiched between capped carbon nanotube junctions
Source: RSC Adv. 2022 Jan 11;12(3):1758–68. doi: 10.1039/d1ra08810f (PMC8979219; doi:10.1039/d1ra08810f)
Supplement: RA-012-D1RA08810F-s001 [file RA-012-D1RA08810F-s001.pdf]

Electronic Supplementary Material (ESI) for RSC Advances.
This journal is © The Royal Society of Chemistry 2021

Supporting information for:

Negative differential resistance behavior of Si/B - substituted into a C_6 chain sandwiched between capped carbon nanotube junctions

Najmeh Janatipour[1], Zabiollah Mahdavifar[*1], Siamak Noorizadeh[1], Georg Schreckenbach[2]

[1]Department of Chemistry, Faculty of Science, Shahid Chamran University of Ahvaz, Ahvaz, Iran

[2]Department of Chemistry, University of Manitoba, Winnipeg, Manitoba, R3T 2N2, Canada

To whom correspondence should be addressed

Fax: ++98-611-3331042

E-mail: z_mahdavifar@scu.ac.ir (Pr. Zabiollah Mahdavifar)

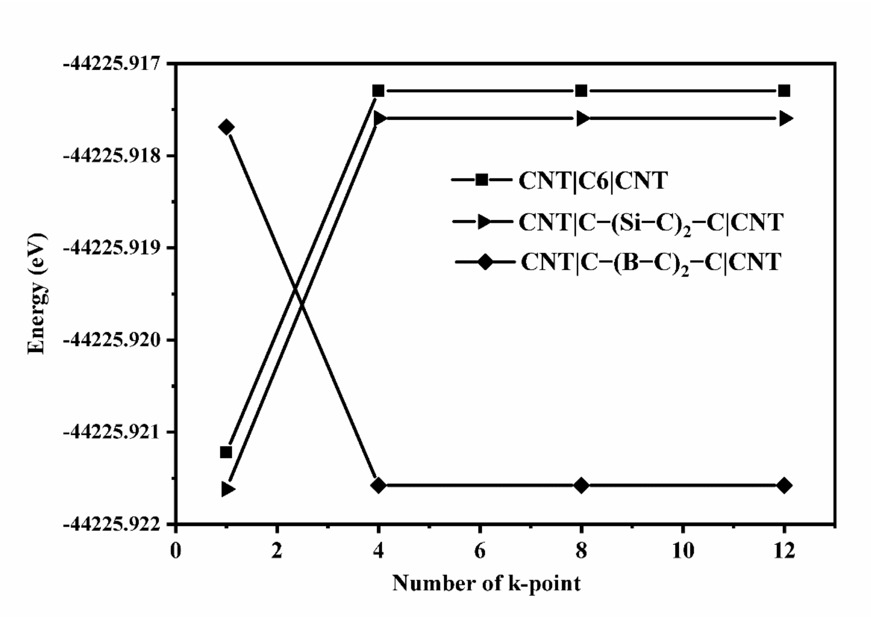

(a)

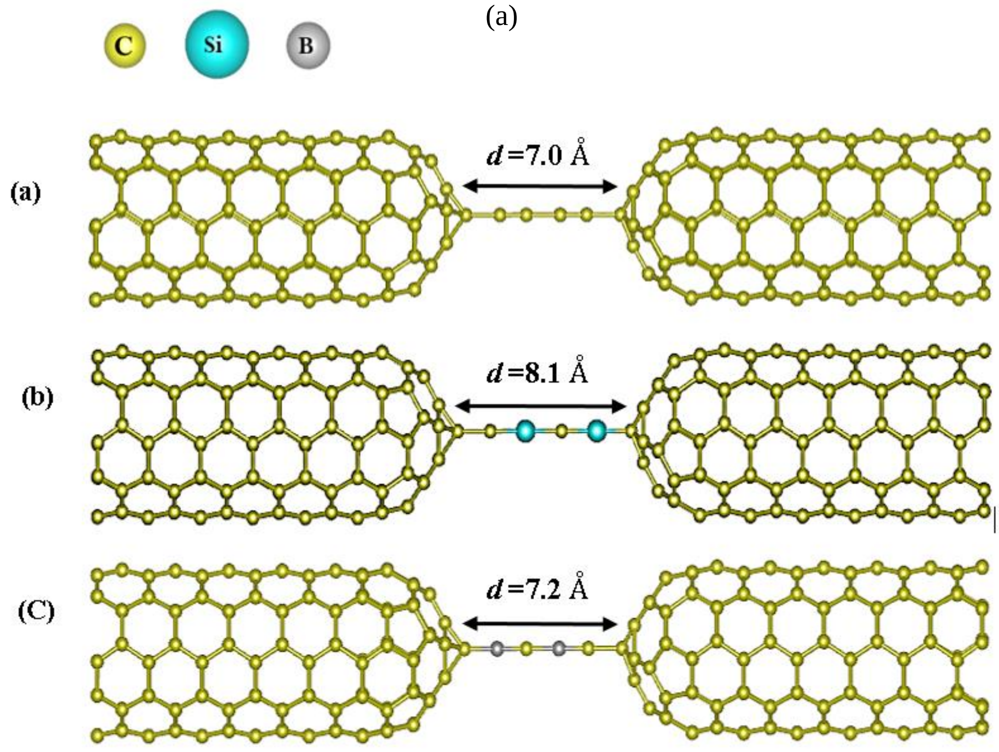

(b)

Fig. S1: (a) Energy (eV) vs. number of k-points for CNT|C6|CNT, CNT|C−(Si−C)$_2$−C|CNT and CNT|C−(B−C)$_2$−C|CNT molecular devices, (b) (b) Initial geometries of (a) C_6, (b) C−(Si−C)$_2$−C and (c) C−(B−C)$_2$−C junctions with CNTs electrodes.

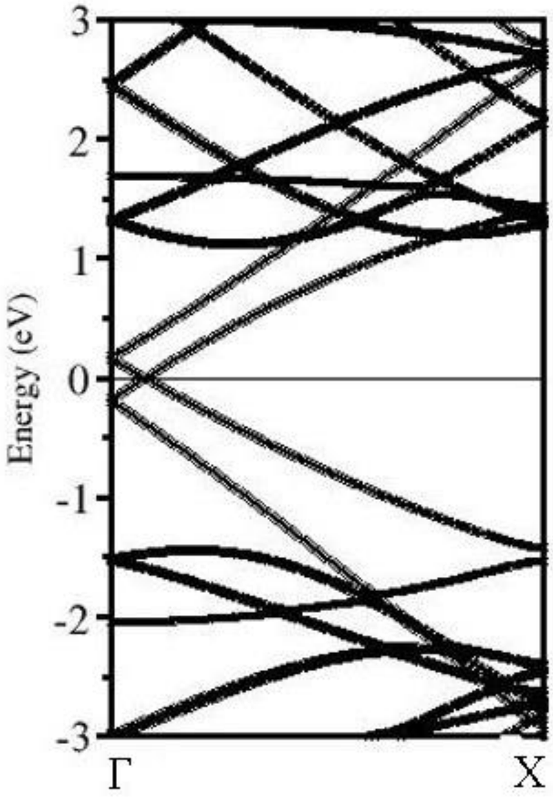

Fig. S2: (a) Band structure of CNT electrodes (including five nanotube lead layers). The Fermi level is set to zero. The solid line represents the Fermi level.

CNT|C6|CNT

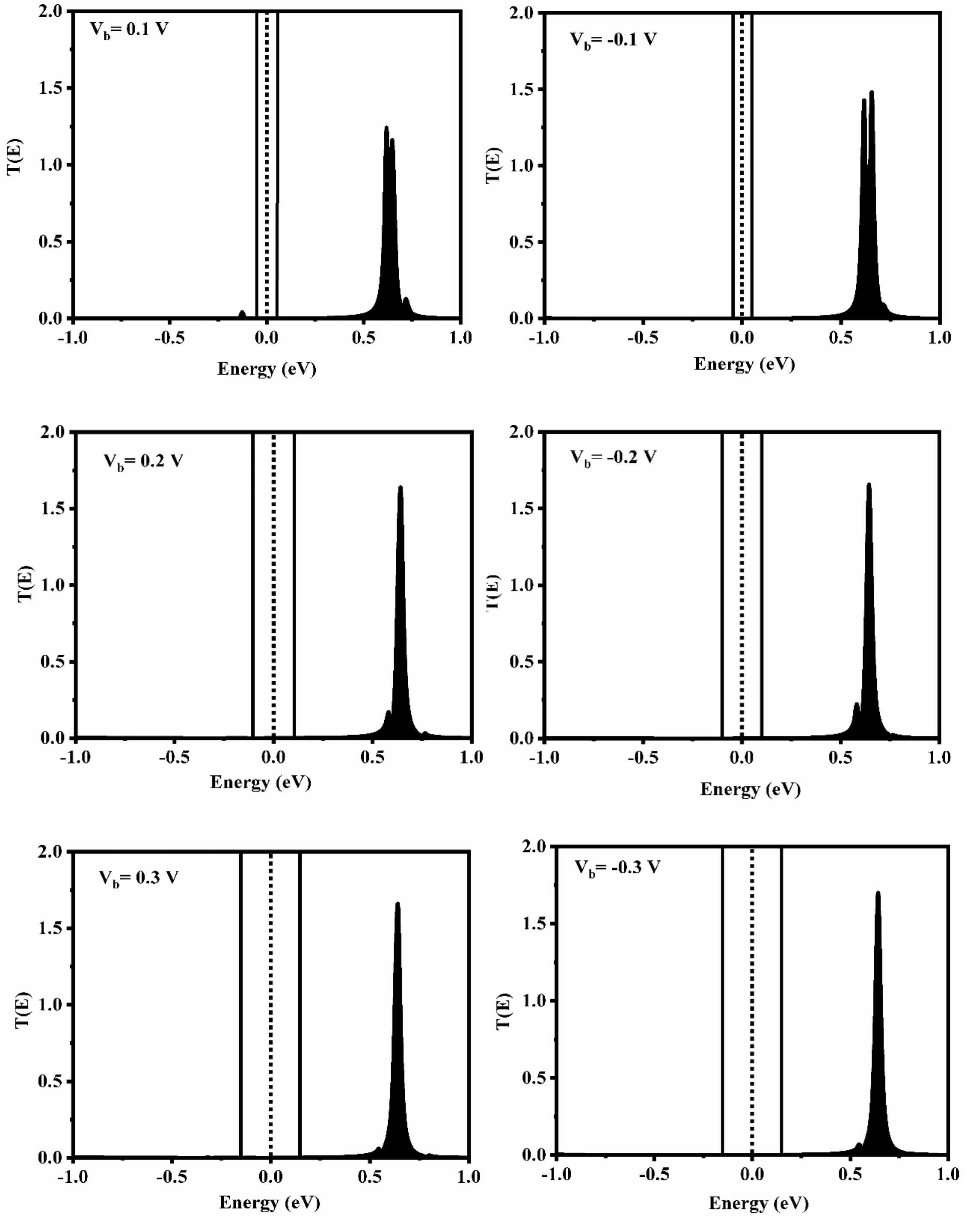

Fig. S3: Bias-dependent transmission spectra of (a) CNT|C_6|CNT, (b) CNT|C−(Si−C)$_2$−C|CNT, and (c) CNT|C−(B−C)$_2$−C|CNT molecular devices. The Fermi level is set to zero. The dotted lines represent the Fermi level. The energy regions between the two solid lines show the bias window.

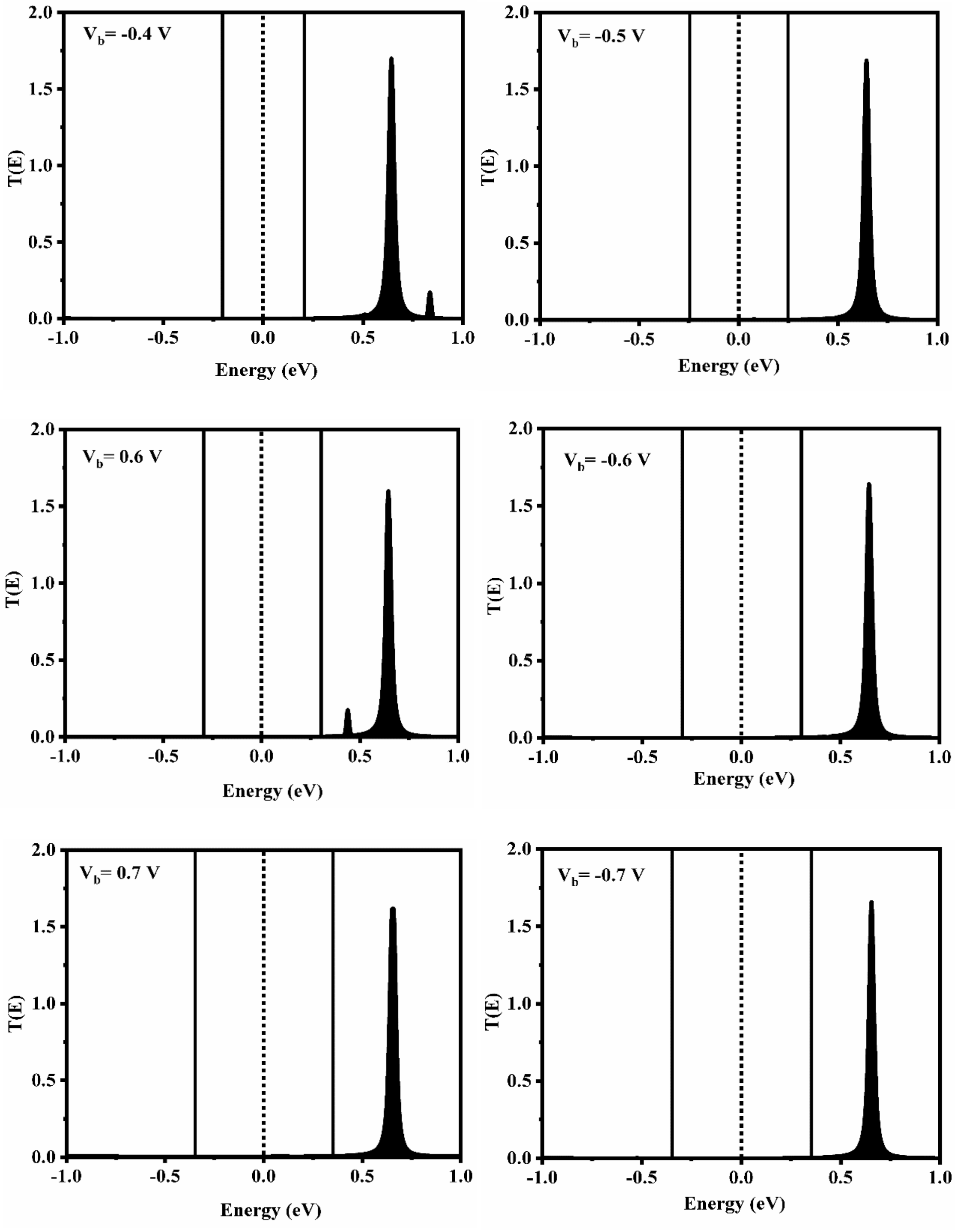

Fig. S3: continued

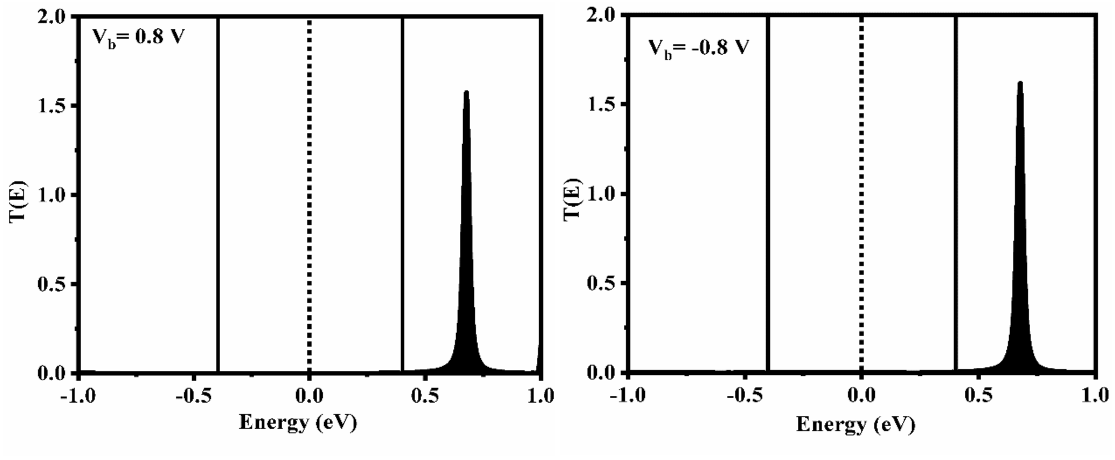

(a) CNT|C−(Si−C)₂−C|CNT

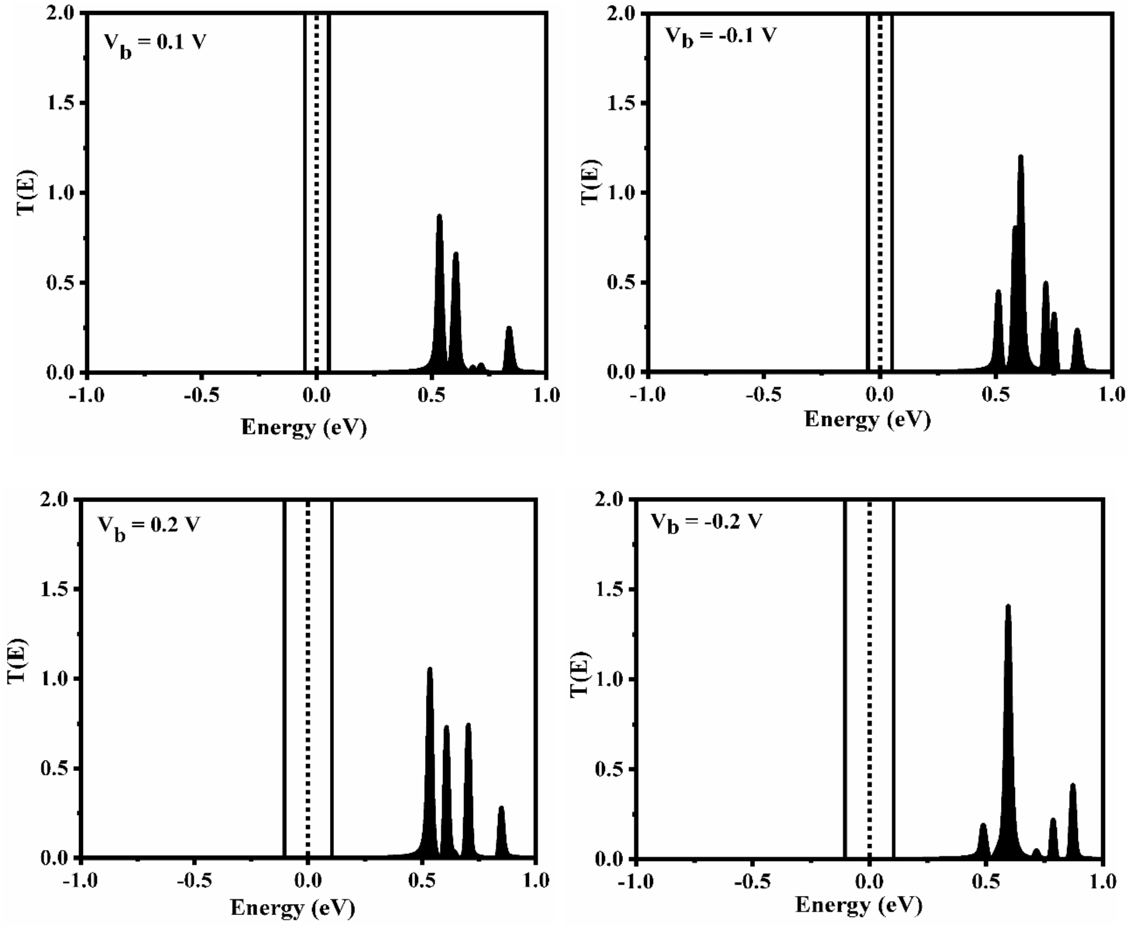

Fig S3: continued

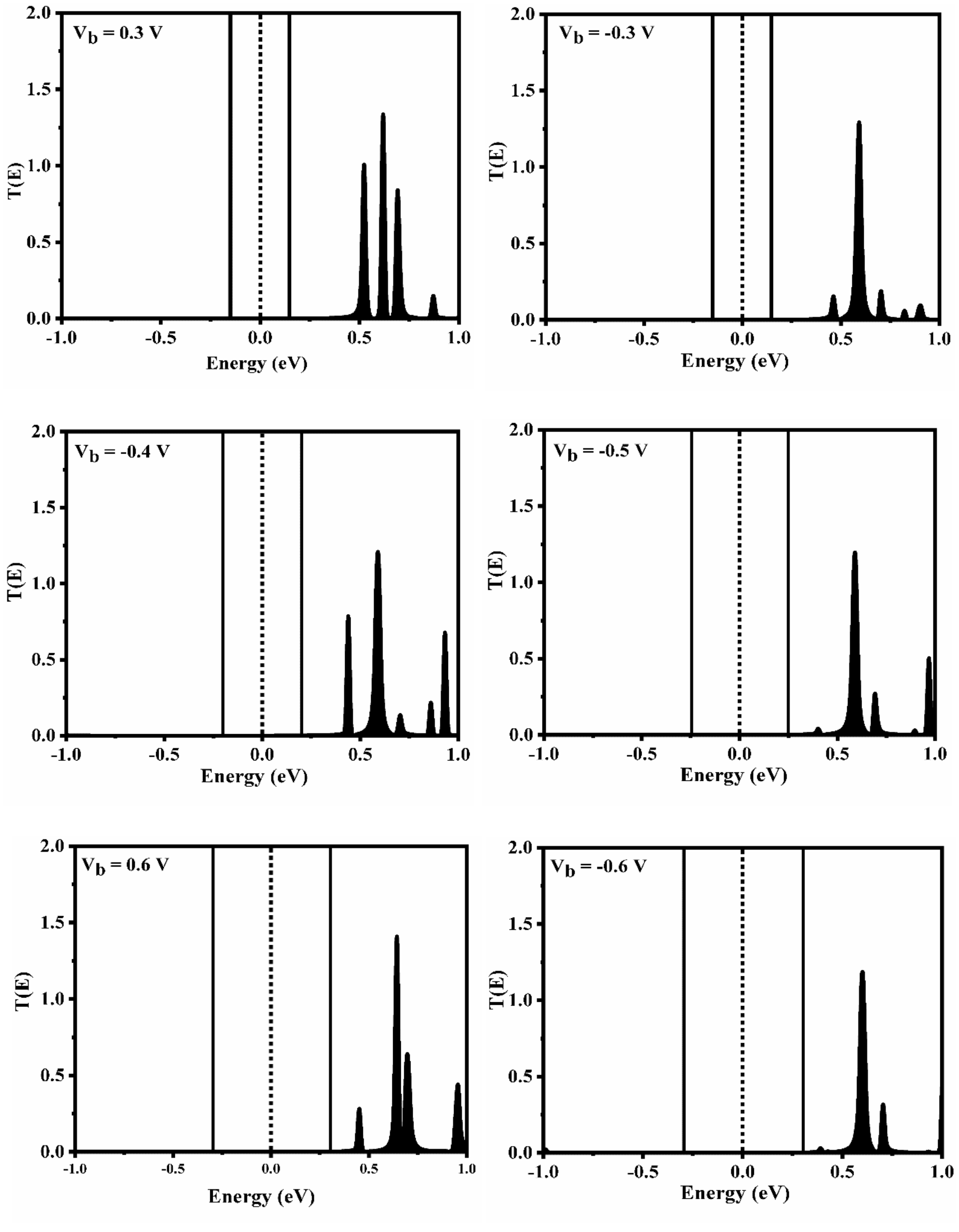

Fig S3: continued

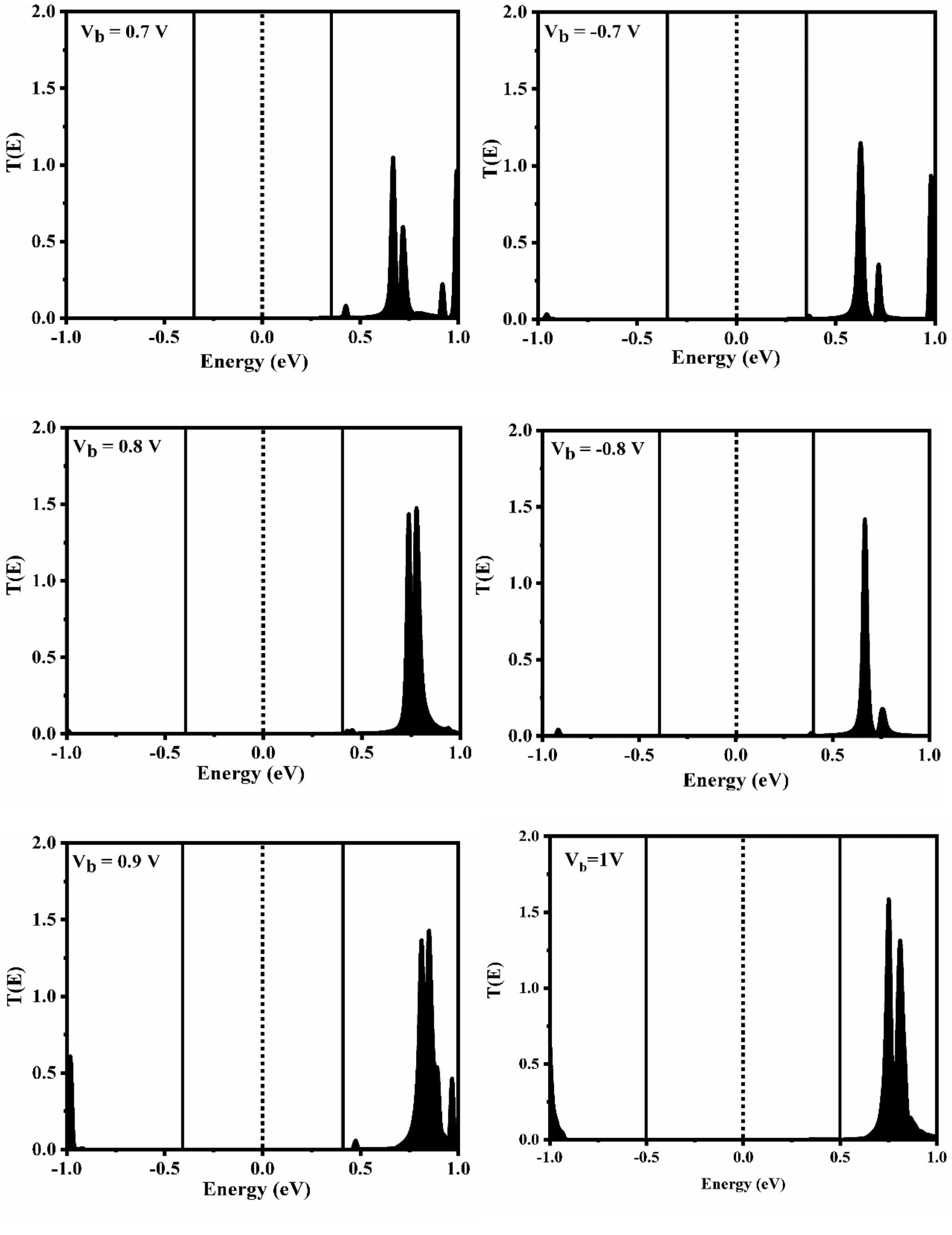

Fig S3: continue.

(b) CNT|C−(B−C)$_2$−C|CNT

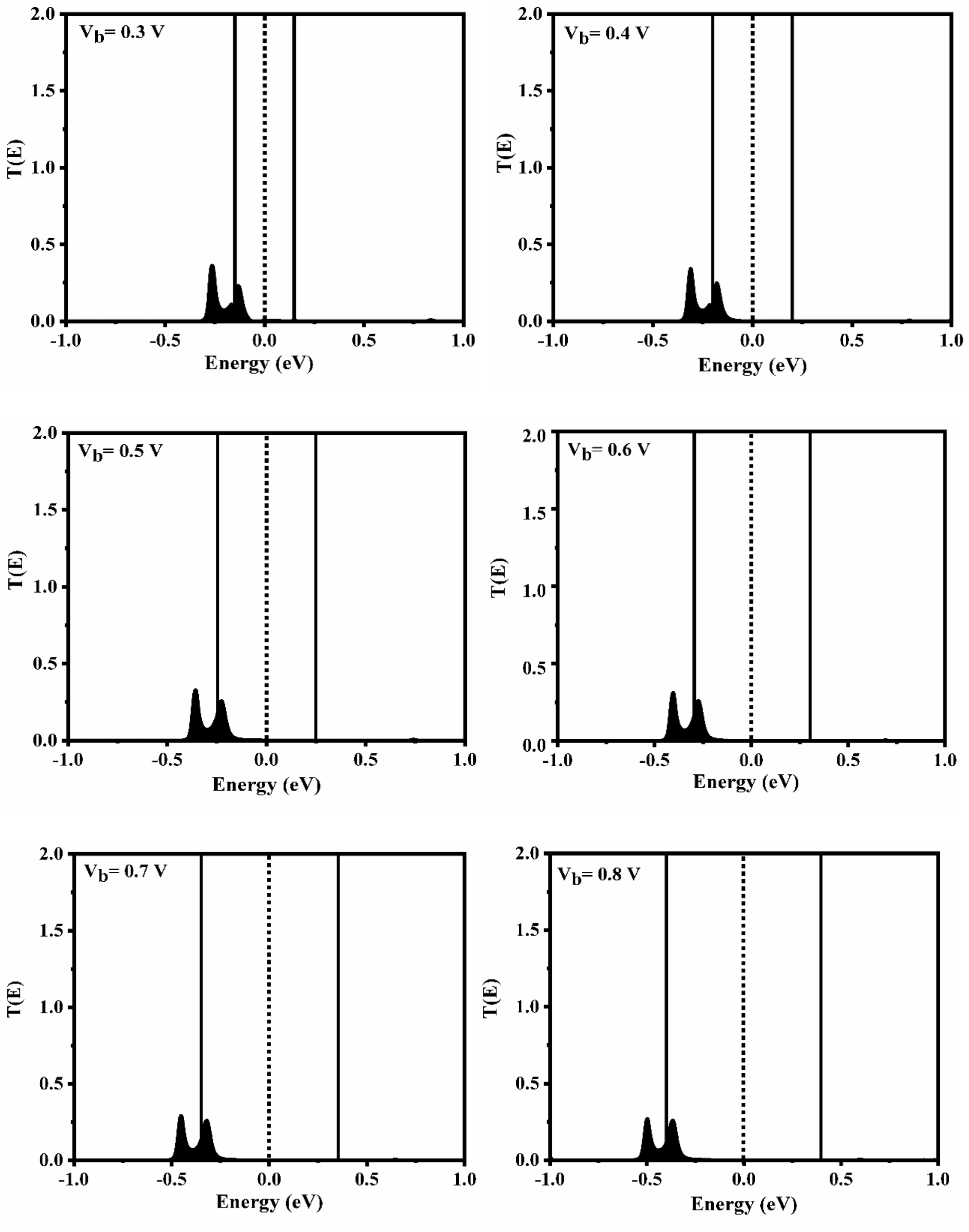

Fig S3: continued

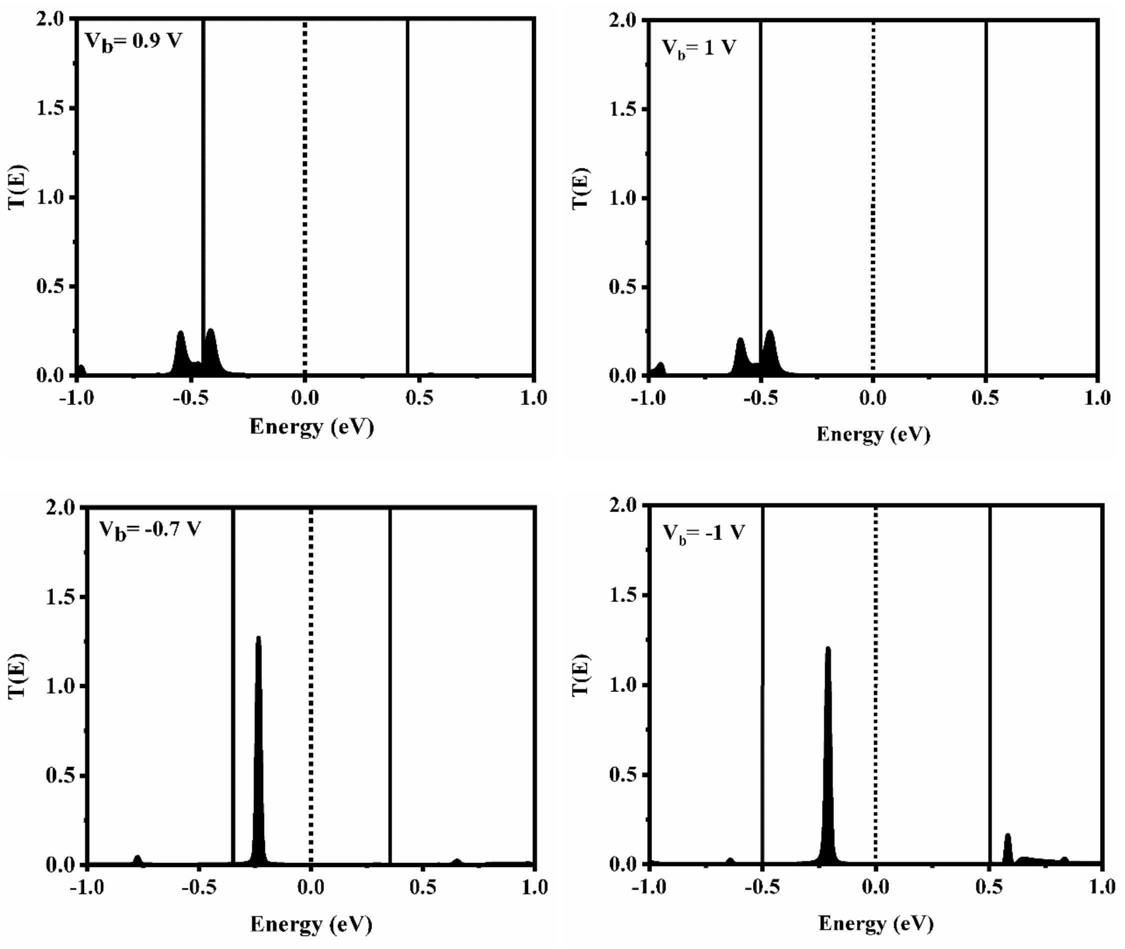

Fig S3: continued
